# A System of Genito-Urinary Diseases; Syphiology and Dermatology

**Published:** 1893-09

**Authors:** 


A SYSTEM OF GENITO-URINARY DISEASES, SYPHIOLOGY AND DERMATOLOGY, by Various Authors. Edited by P. A. Morrow, A.M., M. D. D. Appleton & Co., New York.

It is always a pleasure to us to review good books, and we have taken special pleasure in reviewing the first volume of this valuable system, and if the second and third volumes treat the subjects of syphilis and skin diseases in the same style genito-urinary diseases are treated in this volume, the profession will be under many obligations to both editor and publishers. The articles are written by members of our profession who have given special study to the subjects treated, and they have handled the subjects in a practical way, which will make the work of real value to those who read. We have not seen the subjects of Endoscopy and Cystoscopy handled in so clear a manner in any other work.

The section on gonorrhœa in the female should be read by every physician.

In a short article Dr. W. K. Otis gives very practical directions for the treatment of that most troublesome affection, chronic urethritis, which if followed, will usually give good results, as we can testify, as we have been following about the same plan of treatment for the past two years. But there is no use to specialize, for the whole work is good. J. A. C.

